# Publisher Correction: A pesticide and iPSC dopaminergic neuron screen identifies and classifies Parkinson-relevant pesticides

**DOI:** 10.1038/s41467-023-39001-7

**Published:** 2023-06-23

**Authors:** Kimberly C. Paul, Richard C. Krolewski, Edinson Lucumi Moreno, Jack Blank, Kristina M. Holton, Tim Ahfeldt, Melissa Furlong, Yu Yu, Myles Cockburn, Laura K. Thompson, Alexander Kreymerman, Elisabeth M. Ricci-Blair, Yu Jun Li, Heer B. Patel, Richard T. Lee, Jeff Bronstein, Lee L. Rubin, Vikram Khurana, Beate Ritz

**Affiliations:** 1 Department of Neurology, UCLA David Geffen School of Medicine, Los Angeles, CA USA; 2 Division of Movement Disorders, Department of Neurology, Brigham and Women’s Hospital and Harvard Medical School, Boston, MA 02115 USA; 3 Department of Stem Cell and Regenerative Biology, Harvard University, Cambridge, MA USA; 4 University of Arizona, Mel and Enid Zuckerman College of Public Health, Tucson, AZ USA; 5 UCLA Center for Health Policy Research, Los Angeles, CA USA; 6 Department of Population and Public Health Sciences, Keck School of Medicine, University of Southern California, Los Angeles, CA USA; 7 Harvard Stem Cell Institute, Cambridge, MA USA; 8 Division of Cardiovascular Medicine, Department of Medicine, Brigham and Women’s Hospital and Harvard Medical School, 75 Francis St, Boston, MA 02115 USA; 9 Broad Institute of MIT and Harvard, Cambridge, MA 02142 USA; 10 Department of Epidemiology, UCLA Fielding School of Public Health, Los Angeles, CA USA; 11 Present Address: Prime Medicine, Inc, Cambridge, MA USA; 12 Present Address: Recursion Pharmaceuticals, Salt Lake City, UT USA; 13 Present Address: Nash Family Department of Neuroscience at Mount Sinai, New York, NY USA

**Keywords:** Parkinson's disease, Cell death in the nervous system, Environmental sciences

Correction to: *Nature Communications* 10.1038/s41467-023-38215-z, published online 16 May 2023

In this article the affiliation details for Beate Ritz were incorrectly given as ‘Division of Movement Disorders, Department of Neurology, Brigham and Women’s Hospital and Harvard Medical School, Boston, MA 02115, USA’ and ‘Department of Epidemiology, UCLA Fielding School of Public Health, Los Angeles, CA, USA’ but should have been ‘Department of Neurology, UCLA David Geffen School of Medicine, Los Angeles, CA, USA’ and ‘Department of Epidemiology, UCLA Fielding School of Public Health, Los Angeles, CA, USA’. The original article has been corrected.

