# Supplementary material for: A pesticide and iPSC dopaminergic neuron screen identifies and classifies Parkinson-relevant pesticides
Source: Nat Commun. 2023 May 16;14:2803. doi: 10.1038/s41467-023-38215-z (PMC10188516; doi:10.1038/s41467-023-38215-z)
Supplement: Supplementary file 1 — Supplementary Information [file 41467_2023_38215_MOESM1_ESM.pdf]

SUPPLEMENTARY MATERIALS

Supplementary Methods:

PEG Study Enrollment

For PEG1 patient enrollment, 1,167 potentially eligible patients were identified through large medical groups, neurologists, and public service announcements, 604 did not meet eligibility criteria for the following reasons: 397 had been diagnosed with PD >3 years prior to recruitment, 134 lived outside the tri-counties, and 73 did not have PD. From the 563 remaining potential cases, 90 could not be examined by our movement disorder specialists, 56 declined or moved away, and 34 became too ill or died before the scheduled appointment. Of the 473 examined, 94 did not meet criteria for idiopathic PD, an additional 13 were reclassified as not having PD during follow-up, and 6 participants withdrew after examination and before interview. Of the remaining 360 patients, 357 provided all information necessary for inclusion in this study.

For PEG2 patient enrollment, we were able to draw study participants from the pilot PD registry program in California. This registry builds on a CA law enacted in 2004 to collect and register PD case records from all health care providers. Between 2010-2015, we screened 2,713 potentially eligible PD patients with an address in the tri-county study area reported to the registry. Of these, 397 denied having PD, 212 were diagnosed with PD prior to 2007 (the earliest diagnosis year allowed for enrollment), 39 did not live in the tri-county area, 1,042 were already deceased or too ill, and 293 were unable to be contacted or refused. Of the 730 eligible registry-reported patients, 601 were examined by the UCLA movement disorder specialists, and 126 did not meet criteria for idiopathic PD. Of the remaining 481 patients, 472 provided all information necessary for inclusion in this study.

Population-based controls for both study waves were required to be > 35 years of age, have lived within one of the three counties for at least 5 years before enrollment, and not have a diagnosis of PD. We identified potentially eligible controls initially through Medicare enrollee lists (2001) but mainly from publicly available residential tax-collector records (after 2001 due to HIPAA restrictions). We used two sampling strategies to increase enrollment success and representativeness of the source population: a) for PEG1, random selection from the Medicare enrollee lists and of residential parcels (identified from the tax-collector records) followed by mail or phone enrollment, and b) for PEG2, random selection of clustered households (five per cluster, identified through the tax-collector records) visited in person to enroll at least one eligible control from each cluster (only one per household allowed).

For PEG1, we contacted 1,212 potentially eligible controls. Of these individuals, 457 were ineligible: 409 were < 35 years of age, 44 were too ill to participate, and 4 resided primarily outside the study area. Of the 755 eligible population controls, 409 declined participation, were too ill, or moved before an interview was possible, resulting in the enrollment of 346 population controls. A pilot test mailing, for which the number of eligible participants who declined was not known, enrolled another 62

controls. Of the 408 PEG1 controls, 400 provided all information necessary for inclusion in this study. For PEG2, with the second sampling strategy, 4,756 individuals were screened, of whom 3,515 were ineligible (88% of these were out of the required age range) and 634 declined participation. Of the 607 PEG2 population controls enrolled, 183 completed only an abbreviated interview that assessed the most recent residential and workplace addresses, limiting long-term pesticide exposure assessment, thus, these individuals were excluded. From PEG2, 424 controls provided all information necessary for inclusion in this study.

PEG Pesticide Exposure Assessment

We estimated ambient exposure to specific pesticide active ingredients (AIs) due to living or working near agricultural pesticide application, using record-based pesticide application data and a geographic information systems (GIS)-based model[1].

Since 1974, California law mandates the recording of all commercial agricultural pesticide use by pest control operators and all restricted pesticide use by anyone until 1989, and then (1990-current) all commercial agricultural pesticide use by anyone to the PUR database of the CA-DPR. This database records the location of applications, which can be linked to the Public Land Survey System (PLSS), poundage, type of crop, and acreage a pesticide has been applied on, as well as the method and date of application. The PUR database includes ~5.9 million records for the tri-county area and study period (1974-2017), documenting over 40 years of agricultural pesticide applications. We combined this database with maps of land-use and crop cover, providing a digital representation of historic land-use, to determine the pesticide applications at specific agricultural sites[2]. PEG participants provided lifetime residential and workplace address information, which we geocoded in a multi-step process[3]. For each pesticide in the PUR and each participant, we determined the pounds of pesticide applied per acre within a 500m buffer of each residential and workplace address each year since 1974, weighing the total poundage by the proportion of acreage treated (lbs/acre).

We were interested in long-term ambient exposures, and thus, considered the study exposure window as 1974 to 10 years prior to index date (PD diagnosis for cases or interview for controls), to account for a prodromal PD period. The exposure windows covered a very similar length and temporal period on average for patients and controls of each wave. For PEG1, the mean index year for PD patients was 2001.8 (SD=2.5 years) and 2002.4 (SD=1.6 years) for controls. For PEG2, the mean index year was 2009.2 (SD=3.3 years) for patients and 2009.2 (SD=0.5 years) for controls. This represents on average 22 years of ambient residential exposure information and 18 years of ambient workplace exposure information for study participants, taking the 10-year lag period prior to diagnosis or index date into account and only including years for which the participant reported an address we could geocode (comparisons shown in Supplementary Table 12 and the study windows are detailed in

Supplementary Table 1). To assess exposure across the study window of interest, for each pesticide, we averaged the annual lbs/acre estimates in the study window (e.g. lbs/acre estimates for all 22 years were averaged for participants with 22 years of exposure history). For averaging across the study window, we only used years for which the participants provided address information.

This approach created one summary estimate of the average pounds of pesticide applied per acre per year within the 500m buffer for each pesticide. The summary exposure measure was estimated at residential and workplace locations separately for each participant. In total 722 pesticides had reported application within 500m of at least one PEG participant's residence or workplace. However, we included only 288 chemicals in our PWAS, according to our criterion of having at least 25 study participants with estimated exposure in both the residence and workplace datasets. We log transformed the exposure measure offset by one, centered and scaled the estimates to their standard deviations.

PEG Exposure Assessment Limitations

The exposure assessment method did not account for potentially relevant factors, such as wind patterns at the time of application or geographic features that may influence pesticide drift, and it also assumes that the participant was at the recorded location during the exposure relevant time or that the agent was still active and exposed the residents after application had occurred. However, being within a certain buffer of a pesticide application is one of the strongest predictors of air concentrations of pesticides, and resuspension from these applications is generally constant over at least a week-long period[4]. We have also previously validated our approach with high specificity for organochlorines with serum measurements[5].

Statistical Analysis

We describe our epidemiologic analytic methods and results in four parts: 1) describing the extent of agricultural pesticide application in the study area; 2) the pesticide-wide association analysis; 3) pesticide group overrepresentation analysis; 4) PD-associated pesticide clustering. All analyses were done in R version 4.1.0.

First, we assessed the extent of agricultural pesticide application with basic descriptive statistics (mean, median, range, and standard deviation). We aggregated the PUR-reported total number of different pesticide active ingredients (AI) applied in both the entire study region (tri-counties: Kern, Fresno, and Tulare) and specifically within 500m of PEG participants' residence and workplace locations, and we also aggregated the total reported pounds of pesticide applied. Pesticide products are often composed of one or more AIs, plus any inert ingredients which are generally proprietary and confidential. Thus, the 5.9 million PUR records in the tri-county area do contain information on different pesticide active ingredients applied at the same time or in the same product. However, the summed

pounds of pesticide applied was specific to the AI within the product, and thus the way we aggregated did not over-count by summing the pounds of product applied.

Second, for the pesticide-wide association analysis, we conducted univariate, unconditional logistic regression to calculate odds ratios (ORs) and 95% confidence intervals (CIs) for PD with each pesticide (n=288) individually. Proximity estimated exposures at each location (residence and workplace) were assessed independently and separately for the PEG1 and PEG2 study waves. We combined the OR estimates from the study wave and location stratified analyses in a fixed effects meta-analysis, using a generic inverse-variance method for pooling (R, meta package, metagen function, coding as described Chapter 4.3[6]). We controlled for age, sex, race/ethnicity, education (years of schooling), and index year (year of diagnosis or interview) to account for temporal trends in pesticide use. We used a false discovery rate (FDR) correction to account for multiple testing. We also assessed differences in neighborhood SES based on census information, to account for confounding by SES factors that also vary with location. However, there were no differences in the neighborhood SES factors comparing where the PD patients versus the controls lived or worked. Thus, these factors were not included as covariates to control for confounding. We performed several sensitivity analyses, including adding an indicator for occupational use of pesticides or fertilizers as a covariate in the model, running analyses with the n=183 abbreviated interview controls (see PEG Study Enrollment above), and assessing results stratified by gender. We further assessed statistical interaction by including a gender*pesticide term in the logistic models for pesticides associated with PD at FDR<0.10 in the primary PWAS analysis.

Third, we used overrepresentation analysis (ORA) to test for overrepresentation of pesticide groups (toxicity groups, chemical classes, and use types) in the set of PD-associated pesticides relative to all pesticides we assessed. We linked each of the 288 pesticides included in the PWAS to chemical, regulatory, and toxicity information using publicly available databases. Only 286 of the 288 tested pesticides are included in the ORA, as two pesticides could not be classified into groups. Specifically, ORA tests whether the set of PD-associated pesticides, meaning all pesticides associated with PD (considered at both FDR<0.05 and p<0.05), contains disproportionally more pesticides from a given group than expected given the distribution of the group in all pesticides assessed, tested with Fisher's exact (R coding described Chapter 2.1[7]]). This type of analysis is commonly applied to evaluate gene set overrepresentation. For the ORA, we considered all toxicity groups, classes, and use types that contained at least 3 pesticides from the PD-associated pesticide set. Altogether we assessed 8 toxicity groups, 5 chemical class groups and 9 use type groups, using the FDR<0.05 cut-off for the PD-associated pesticides, and 8 toxicity groups, 9 chemical classes, and 10 use type groups, p<0.05 cut-off, which are listed in the result tables (Supplementary Table 5).

Fourth, we assessed clustering of the PD-associated pesticides identified by logistic regression (FDR<0.10). We assessed pairwise-correlation between all the PWAS-implicated pesticides using Pearson correlation (Supplementary Tables 9 and 10). In order to assess how the mDA toxic pesticides correlated with the other PWAS-implicated pesticides, we used a data-driven integration and network analysis approach to assess correlations across two layers: the set of mDA toxic pesticides and the set of all other PWAS-implicated pesticides. We assessed Pearson correlation between pesticides across layers at R>0.45 and showed the correlations in a circle graph using Cytoscape. Cytoscape was also used to determine network connectivity measures for each node (pesticide) in the graph, including the closeness centrality and number of edges. We also performed hierarchical Pearson correlation clustering analysis, separately for exposures at residential and workplace addresses, and provided a dendrogram with 1-R (Pearson) as the distance. As pesticides are regularly applied to the same field within the same season, year after year, this analysis assessing exposure clustering for PD-associated pesticides helps highlight real-world co-applications or mixtures and generates co-exposure profiles.

PWAS Pesticides

Two chemicals, DPR codes 752 and 1752, had PUR-reported use near enough PEG study participants to be considered for the analysis (Supplementary Table 2), and thus were included in the initial PWAS analysis. However, although both pesticides showed association with PD in the epidemiologic study, neither pesticide was included in any CA DPR chemical ingredient databases (current or archival) or PAN pesticide information databases. Thus, as the chemical identities could not be determined, these pesticides were removed from any further analysis. The DPR code and associations for these two pesticides from the initial PWAS as described above are provided below.

CA DPR Code	Meta OR	LCL	UCL	FDR	Beta	SE	P-value
Chem752	1.130	1.049	1.217	0.016	0.122	0.038	0.001
Chem1752	1.093	1.009	1.183	0.138	0.089	0.041	0.029

Supplementary Figures:

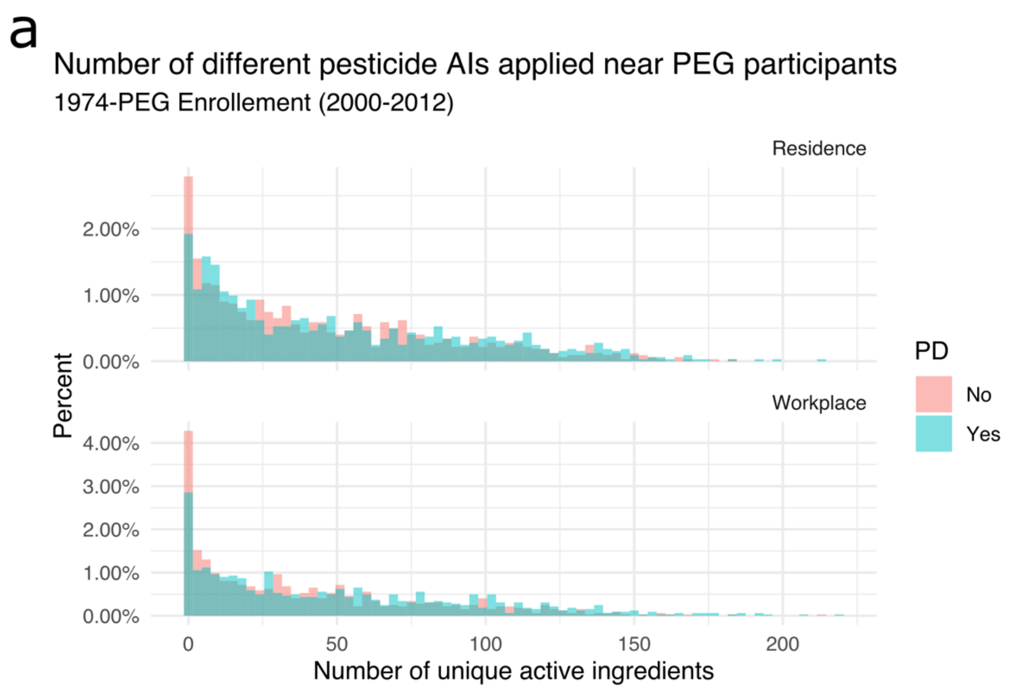

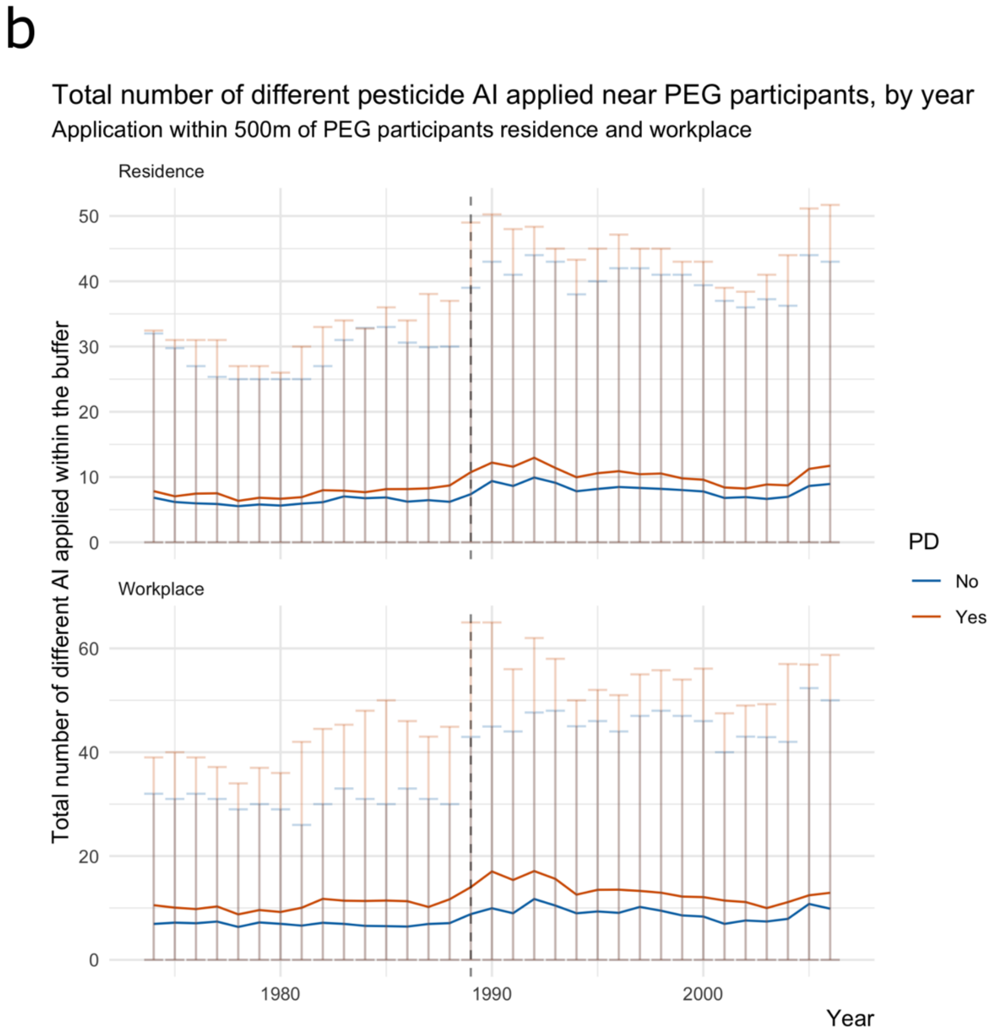

Supplementary Figure 1. Total number of pesticide active ingredients applied near the PEG participants residence and workplace addresses (a) across the study window and (b) by year.

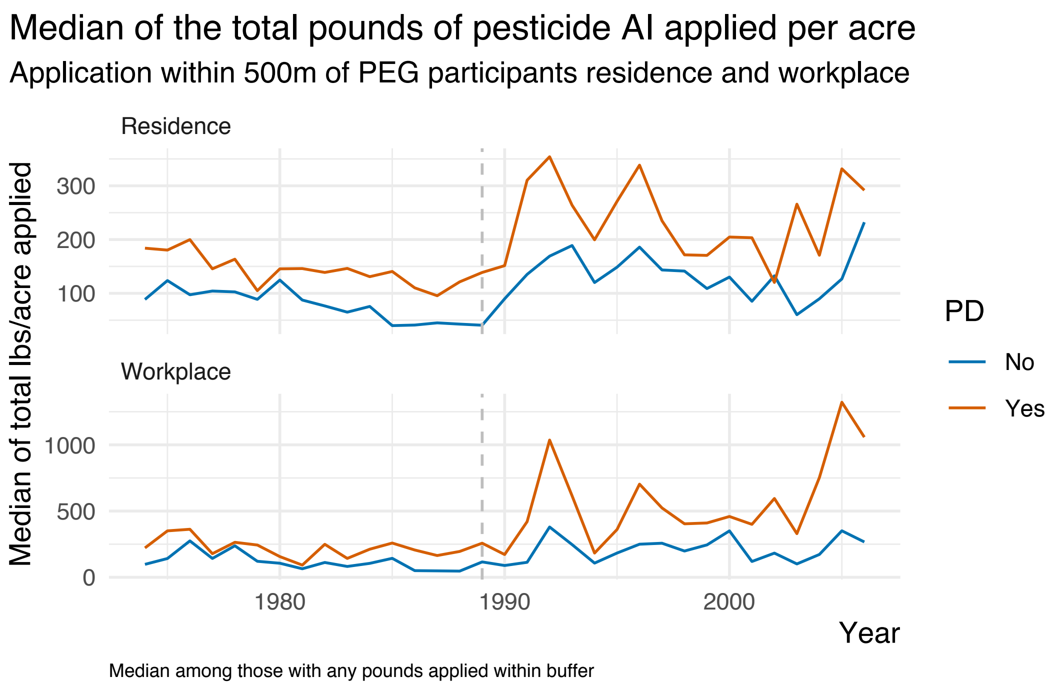

Supplementary Figure 2. Median total pounds of pesticide active ingredient applied per acre near the PEG participants residence and workplace addresses.

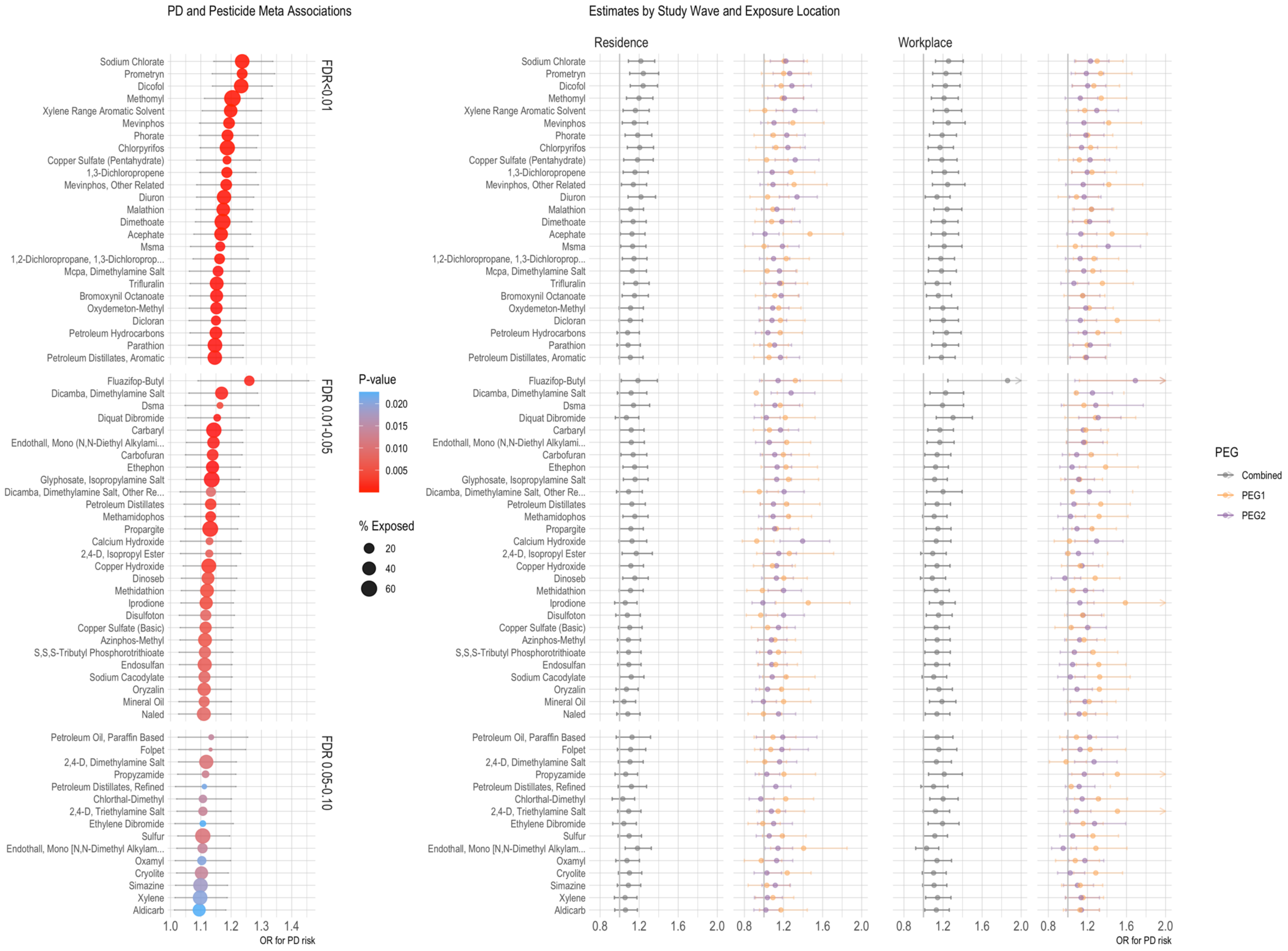

PD and Pesticide Meta Associations
Estimates by Study Wave and Exposure Location
Residence
Workplace
FDR<0.01
FDR 0.01-0.05
FDR 0.05-0.10
Sodium Chlorate
Prometryn
Dicofol
Methomyl
Xylene Range Aromatic Solvent
Mevinphos
Phorate
Chlorpyrifos
Copper Sulfate (Pentahydrate)
1,3-Dichloropropene
Mevinphos, Other Related
Diuron
Malathion
Dimethoate
Acephate
Msma
1,2-Dichloropropane, 1,3-Dichloroprop...
Mcpa, Dimethylamine Salt
Trifluralin
Bromoxynil Octanoate
Oxydemeton-Methyl
Dicloran
Petroleum Hydrocarbons
Parathion
Petroleum Distillates, Aromatic
Fluazifop-Butyl
Dicamba, Dimethylamine Salt
Dsma
Diquat Dibromide
Carbaryl
Endothall, Mono (N,N-Diethyl Alkylami...
Carbofuran
Ethephon
Glyphosate, Isopropylamine Salt
Dicamba, Dimethylamine Salt, Other Re...
Petroleum Distillates
Methamidophos
Propargite
Calcium Hydroxide
2,4-D, Isopropyl Ester
Copper Hydroxide
Dinoseb
Methidathion
Iprodione
Disulfoton
Copper Sulfate (Basic)
Azinphos-Methyl
S,S,S-Tributyl Phosphorotrithioate
Endosulfan
Sodium Cacodylate
Oryzalin
Mineral Oil
Naled
Petroleum Oil, Paraffin Based
Folpet
2,4-D, Dimethylamine Salt
Propyzamide
Petroleum Distillates, Refined
Chlorthal-Dimethyl
2,4-D, Triethylamine Salt
Ethylene Dibromide
Sulfur
Endothall, Mono [N,N-Dimethyl Alkylam...
Oxamyl
Cryolite
Simazine
Xylene
Aldicarb
1.0 1.1 1.2 1.3 1.4
OR for PD risk
P-value
0.020
0.015
0.010
0.005
% Exposed
20
40
60
0.8 1.2 1.6 2.0
OR for PD risk
PEG
Combined
PEG1
PEG2

Supplementary Figure 3. Dot plot displaying the odds ratio (dot; OR) and 95% CI (error bar) from the meta-analysis for all pesticides with an FDR<0.10, as well as results stratified by exposure location and PEG study wave. We used univariate, unconditional logistic regression to calculate odds ratios (ORs) and 95% confidence intervals (CIs) for PD with each pesticide (n=288). We combined the OR estimates from each study wave and location (residential and occupational addresses) in a fixed effects meta-analysis, these results are shown here. P-values were based on a z-score statistic and two-sided interval. P-values were adjusted for multiple testing using an FDR, which are shown in Supplementary Table 3. The log odds ratio is the center of the 95% CI on the logarithmic scale. The log odds ratio and 95% CI on the logarithmic scale were exponentiated to get the odds ratio and 95% CI. Analysis based on n=829 PD patients and n=824 controls.

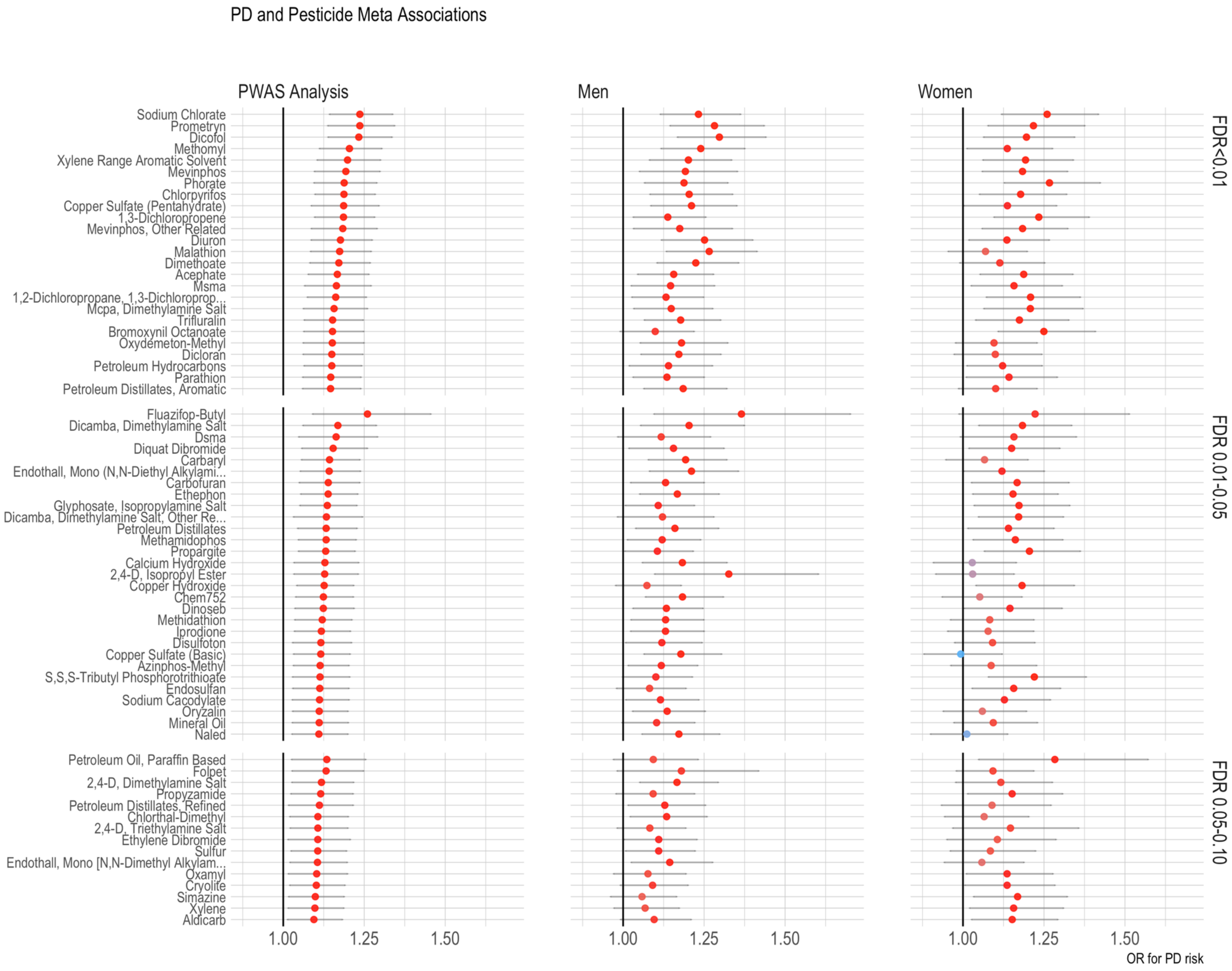
PD and Pesticide Meta Associations
PWAS Analysis
Men
Women
FDR<0.01
FDR 0.01-0.05
FDR 0.05-0.10
Sodium Chlorate
Prometryn
Dicofol
Methomyl
Xylene Range Aromatic Solvent
Mevinphos
Phorate
Chlorpyrifos
Copper Sulfate (Pentahydrate)
1,3-Dichloropropene
Mevinphos, Other Related
Diuron
Malathion
Dimethoate
Acephate
Msma
1,2-Dichloropropane, 1,3-Dichloroprop...
Mcpa, Dimethylamine Salt
Trifluralin
Bromoxynil Octanoate
Oxydemeton-Methyl
Dicloran
Petroleum Hydrocarbons
Parathion
Petroleum Distillates, Aromatic
Fluazifop-Butyl
Dicamba, Dimethylamine Salt
Dsma
Diquat Dibromide
Carbaryl
Endothall, Mono (N,N-Diethyl Alkylami...
Carbofuran
Ethephon
Glyphosate, Isopropylamine Salt
Dicamba, Dimethylamine Salt, Other Re...
Petroleum Distillates
Methamidophos
Propargite
Calcium Hydroxide
2,4-D, Isopropyl Ester
Copper Hydroxide
Chem752
Dinoseb
Methidathion
Iprodione
Disulfoton
Copper Sulfate (Basic)
Azinphos-Methyl
S,S,S-Tributyl Phosphorotrithioate
Endosulfan
Sodium Cacodylate
Oryzalin
Mineral Oil
Naled
Petroleum Oil, Paraffin Based
Folpet
2,4-D, Dimethylamine Salt
Propyzamide
Petroleum Distillates, Refined
Chlorthal-Dimethyl
2,4-D, Triethylamine Salt
Ethylene Dibromide
Sulfur
Endothall, Mono [N,N-Dimethyl Alkylam...
Oxamyl
Cryolite
Simazine
Xylene
Aldicarb
1.00
1.25
1.50
OR for PD risk

Supplementary Figure 4. Dot plot displaying the meta-analysis odds ratio (OR; dot) and 95% CI (error bar) for all pesticides with an FDR<0.10 (primary PWAS analysis) as well as results stratified by sex. Estimates shown are meta-analysis results across study waves and exposure locations. We used univariate, unconditional logistic regression to calculate odds ratios (ORs) and 95% confidence intervals (CIs) for PD with each pesticide. The FDR groupings labeled on the far right are based on the "PWAS Analysis" FDR. The log odds ratio is the center of the 95% CI on the logarithmic scale. The log odds ratio and 95% CI on the logarithmic scale were exponentiated to get the odds ratio and 95% CI. Analysis based on n=829 PD patients (305 women & 524 men) and n=824 controls (441 women & 383 men).

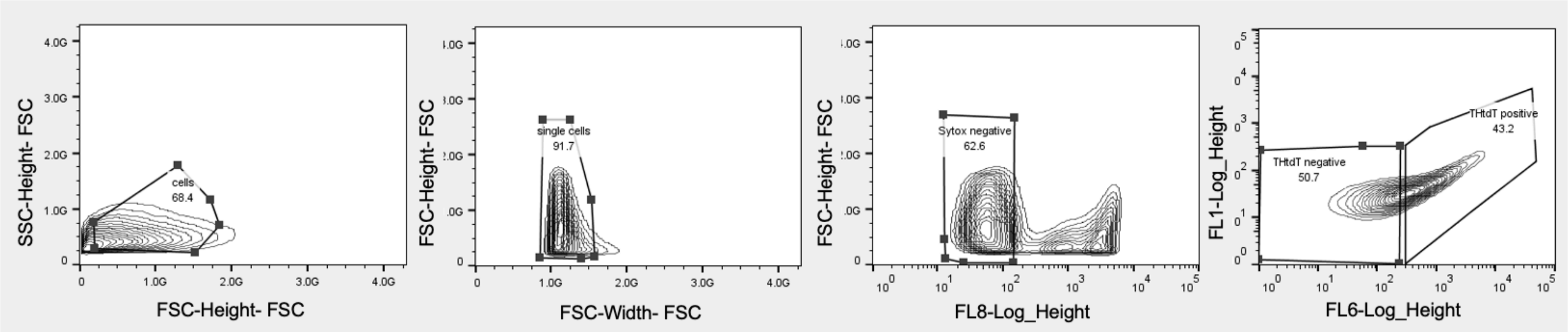

Supplementary Figure 5. Sequential FACS gating strategy from left to right. Sytox Red (Invitrogen) was used to exclude dead cells from the analysis and brightly THtdT positive cells were sorted for downstream analysis. This is a representative single experiment to demonstrate the gating strategy for FACS utilized in live imaging experiments.

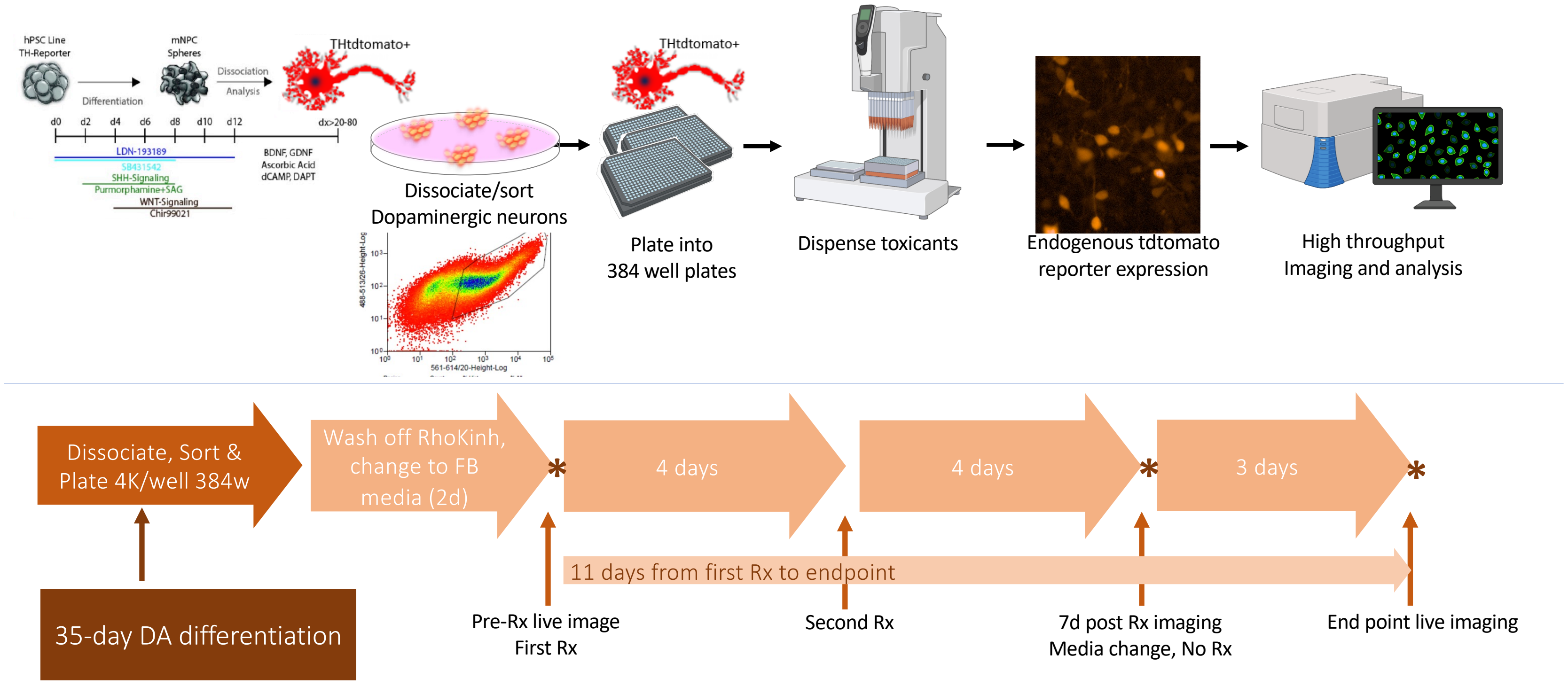

Supplementary Figure 6. Direct treatment of mDA neurons and live imaging schematic. *Upper*: Assay design for custom PWAS in vitro dopaminergic neuron screen. Embryoid bodies/spheres were differentiated with a midbrain patterning protocol. Spheres containing THtdTomato+ dopamine neurons are dissociated for FACS between day 35-42 to purify THtdTomato+ cells which are then plated into multiwell assay plates using an Apricot Personal Pipettor, which was also utilized for media changes and toxicant treatment. Endogenous THtdTomato fluorescence was imaged on an IXM high content microscope fitted with a live cell chamber. *Lower:* timeline and sequencing of treatments, media changes, and imaging. Asterisks show imaging time points. Illustrations made in ©BioRender - biorender.com

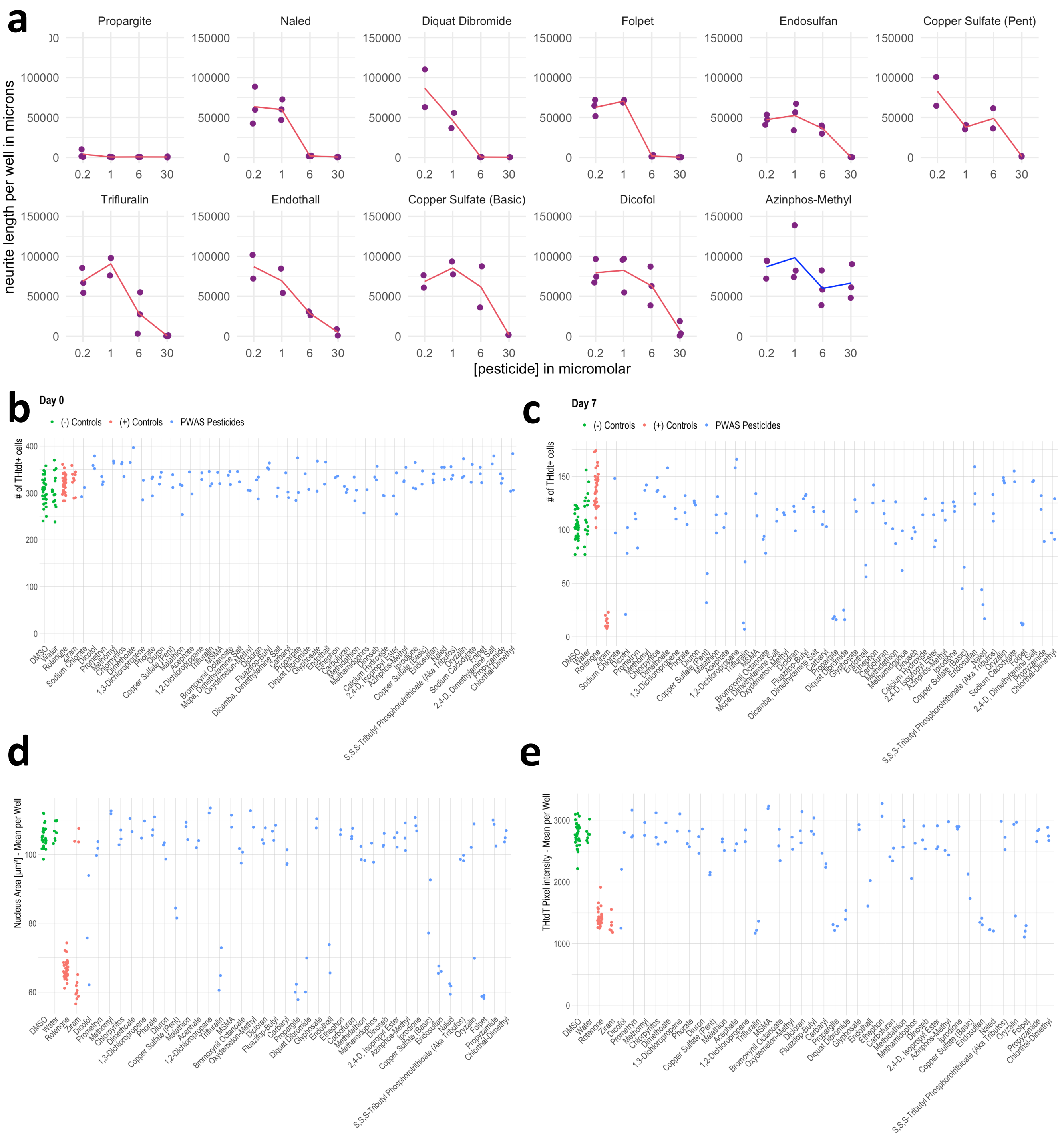

Supplementary Figure 7. Detailed morphometric data from live-imaging PWAS screen. **a**. Dose response curves of neurite length (y-axis) for each of the ten toxic PWAS pesticides. Red lines are average of the data points for each dose. **b,c**. Scatter plots of mDA neuron cell counts are provided for pre-treatment (day 0) and day 7 post treatment. In **b**, the conditions labelled on the x-axis represent the wells that have not yet been exposed to the pesticides listed. The data points depicted here are a series of repeated measures with 7b showing the first measurement, 7c showing the second measurement at day 7 post treatment and main manuscript figure 4a showing the final measurement at day 11 post treatment. **d**. Cell area measurements for the entire set of screened PWAS pesticides. THtdT reporter signal was measured in the cell body excluding signal in the neurites. **e**. Average THtdT signal intensity in each pesticide condition. All data derived from the screen in this figure are from an n = 1 experiment with two or three technical replicates per pesticide.

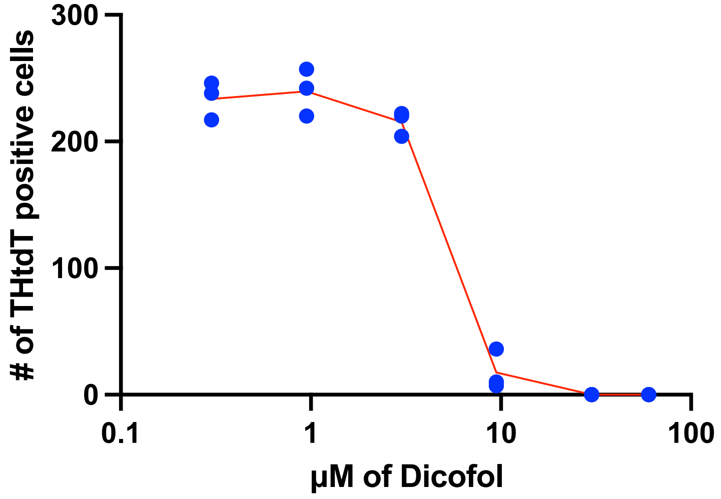

Supplementary Figure 8. Dose response curve confirms toxicity of dicofol. X-axis is concentration of dicofol in micromolar. Red line depicts the average of individual data points at each concentration. Three technical replicates are shown by separate data points from one biological replicate.

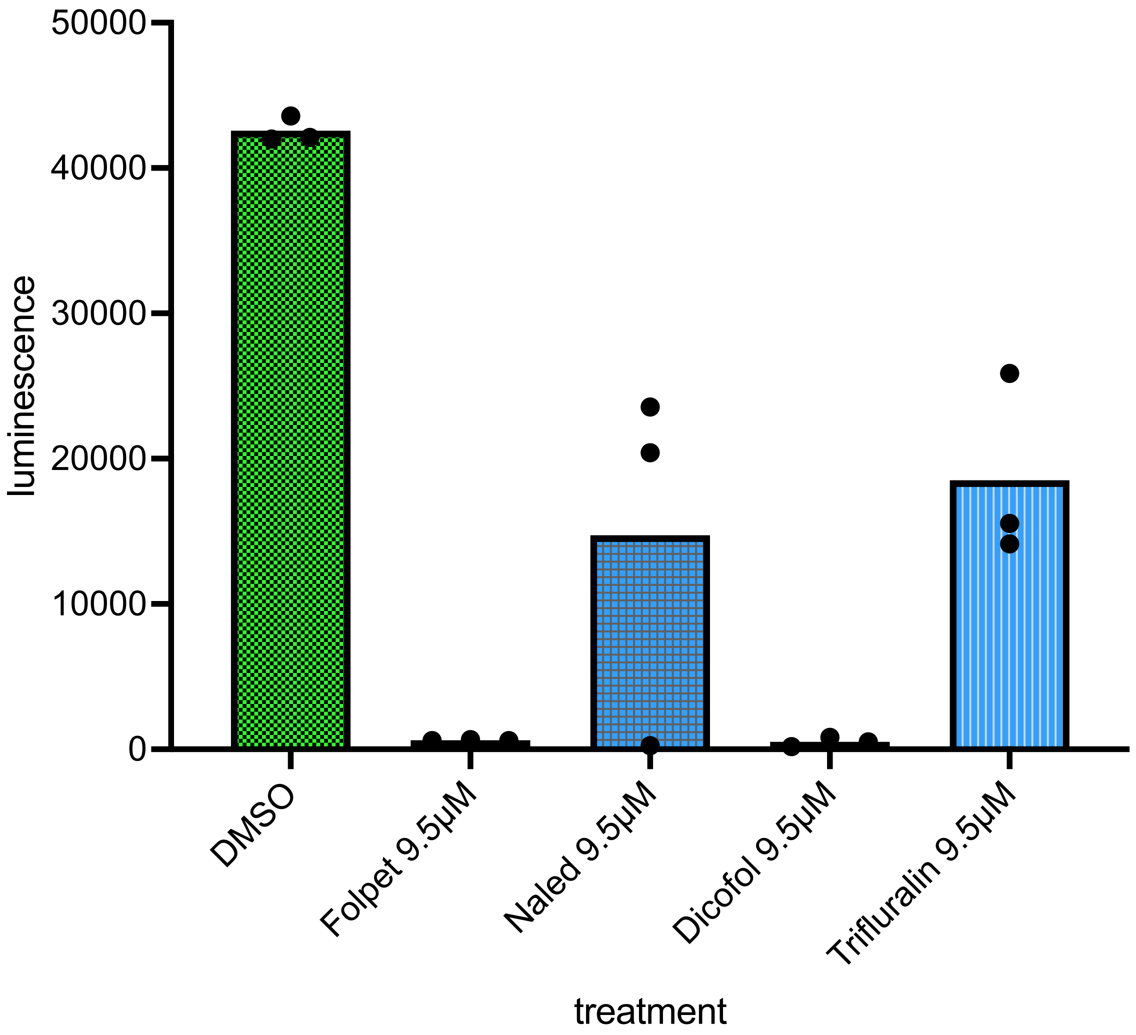

Supplementary Figure 9. Cell viability as assessed by CellTitre-Glo based ATP measurement. The subset of toxic pesticides that are DMSO-soluble were retested in the SNCA triplication THtdT mDA neurons using the CellTitre-Glo assay (Promega) as a readout of remaining viable cells. All four pesticides resulted in the expected reduction of viable cells at or below the original screening concentration as measured by CellTitre-Glo assay at 11 days post pesticide treatment. Average and standard deviation of 2-3 technical replicates from a single experiment are shown.

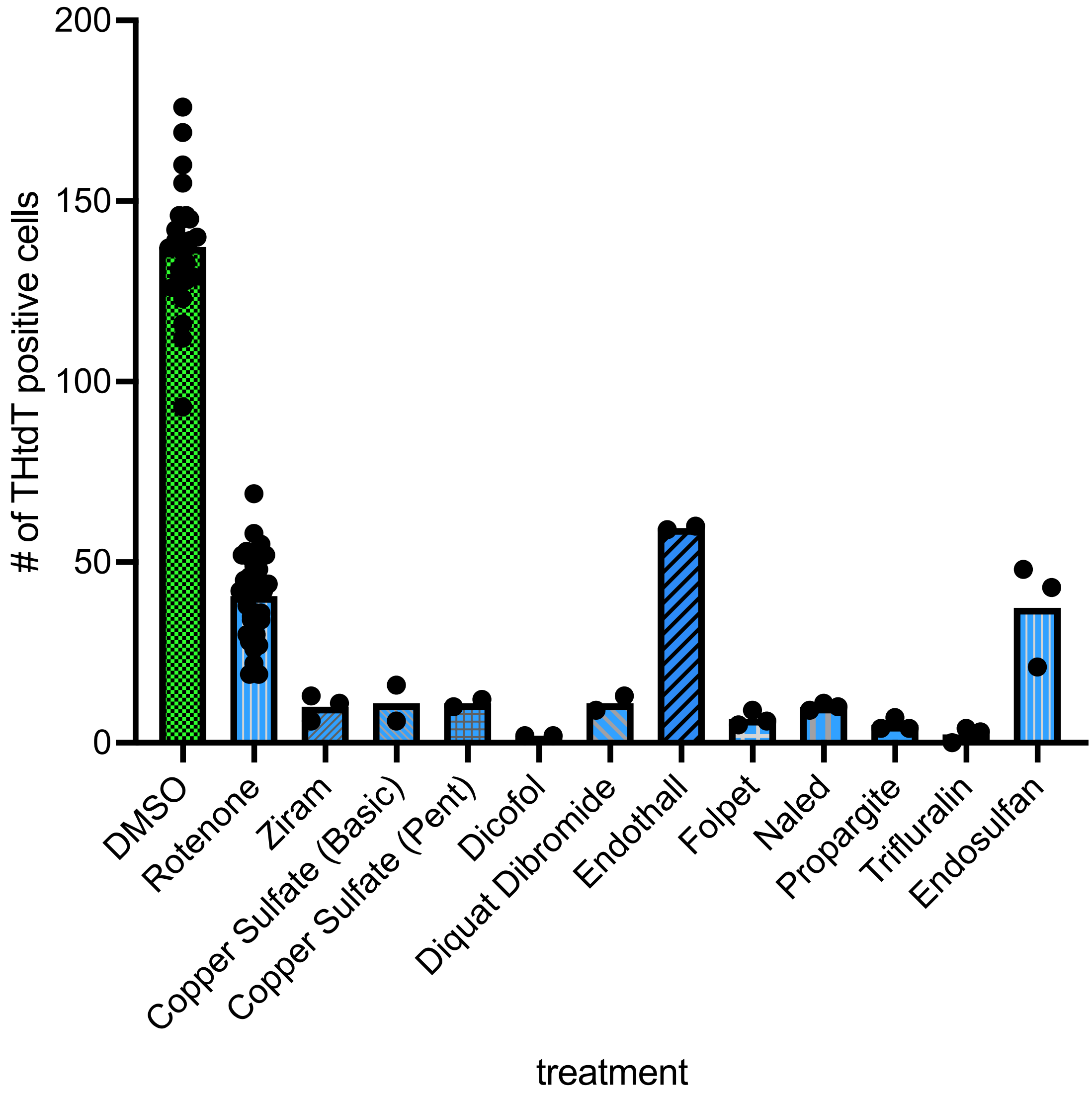

Supplementary Figure 10. Toxicity of pesticides in mDA neurons from an iPSC line with two copies of wild-type α-synuclein. Pesticides identified in the original live imaging assay using sorted mDA neurons from the SNCA triplication cell line were assessed in mDA neurons produced from a cell line with two copies of wild type α-synuclein. The THtdT reporter was inserted into this line to facilitate live image quantitation. Sorted mDA neurons were utilized in an experimental workflow and assay timeline identical to that of Figure 4. For each condition, two to three technical replicates from a single experiment are shown with one standard deviation above and below the mean.

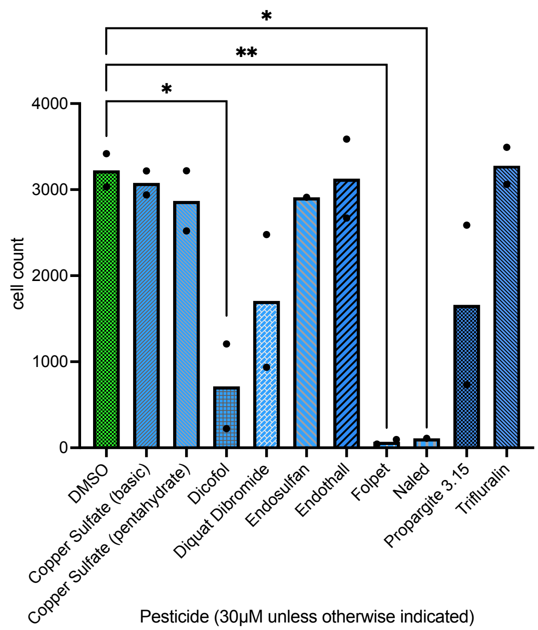

Supplementary Figure 11. Measures of cardiomyocyte toxicity following treatment with PWAS pesticides. SNCA triplication iPSCs were differentiated into cardiomyocytes. Cell counts were measured via Hoechst staining of live nuclei eleven days after the first pesticide treatment. * $p<0.05$; ** $p < 0.01$ by Dunnett's multiple comparisons test. N =2 for cell counts. P-values for comparisons to DMSO are as follows: dicofol p = 0.034; folpet p = 0.009; naled p = 0.0317

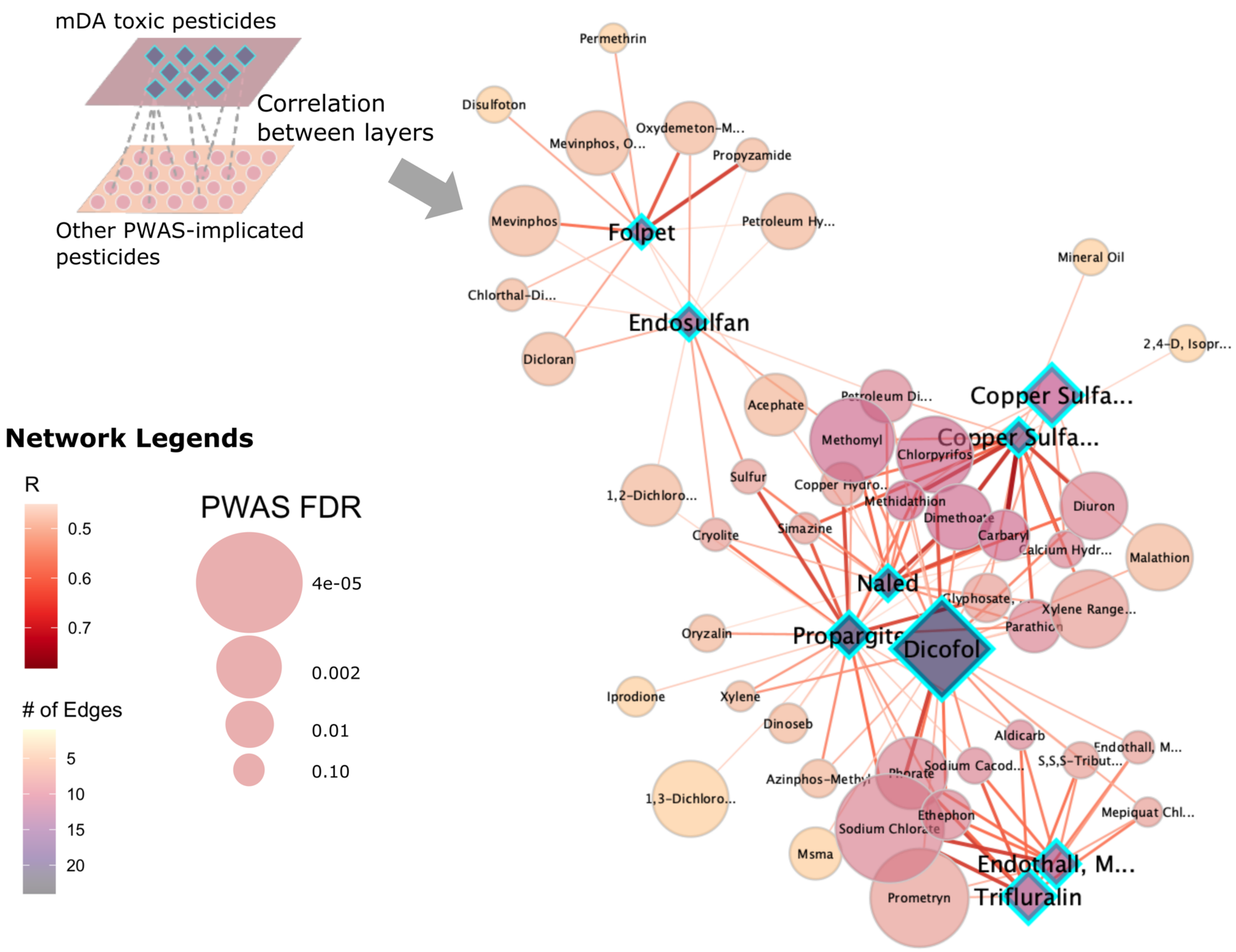

mDA toxic pesticides
Correlation between layers
Other PWAS-implicated pesticides
Network Legends
R
0.5
0.6
0.7
PWAS FDR
4e-05
0.002
0.01
0.10
# of Edges
5
10
15
20
Permethrin
Disulfoton
Oxydemeton-M...
Mevinphos, O...
Propyzamide
Mevinphos
Folpet
Petroleum Hy...
Chlorthal-Di...
Endosulfan
Dicloran
Mineral Oil
2,4-D, Isopr...
Acephate
Copper Sulfa...
Methomyl
Chlorpyrifos
Copper Sulfa...
1,2-Dichloro...
Sulfur
Methidathion
Diuron
Dimethoate
Cryolite
Simazine
Carbaryl
Calcium Hydr...
Malathion
Naled
Glyphosate, ...
Xylene Range...
Oryzalin
Propargite
Parathion
Dicofol
Iprodione
Xylene
Dinoseb
Aldicarb
Endothall, M...
S,S,S-Tribut...
Sodium Cacod...
Azinphos-Methyl
Phorate
1,3-Dichloro...
Ethephon
Mepiquat Chl...
Sodium Chlorate
Msma
Endothall, M...
Trifluralin
Prometryn

Supplementary Figure 12. Correlation network showing the pesticide exposure correlations across two layers: first, the set of mDA neurotoxic pesticides, which are designated as teal highlighted diamonds, and second, the set of all other PWAS-implicated pesticides, shown as circles. Correlations between layers at R>0.45 are shown. The size of the shapes in the correlation circle (diamonds and circles) were determined by the PWAS FDR, so pesticides that were more strongly associated with PD in the PWAS are represented by larger sized shapes. The color of the shapes reflects the density of the connections (i.e. correlations at R>0.45) made by that specific pesticide with others. Pesticides with a darker color are correlated with more pesticides, and arrangement around the circle is ordered from those with the most correlations (dicofol, darkest color) to the least (petroleum hydrocarbons, lightest color). This is an alternate network view as Figure 5b. Source data is the same as Figure 5b.

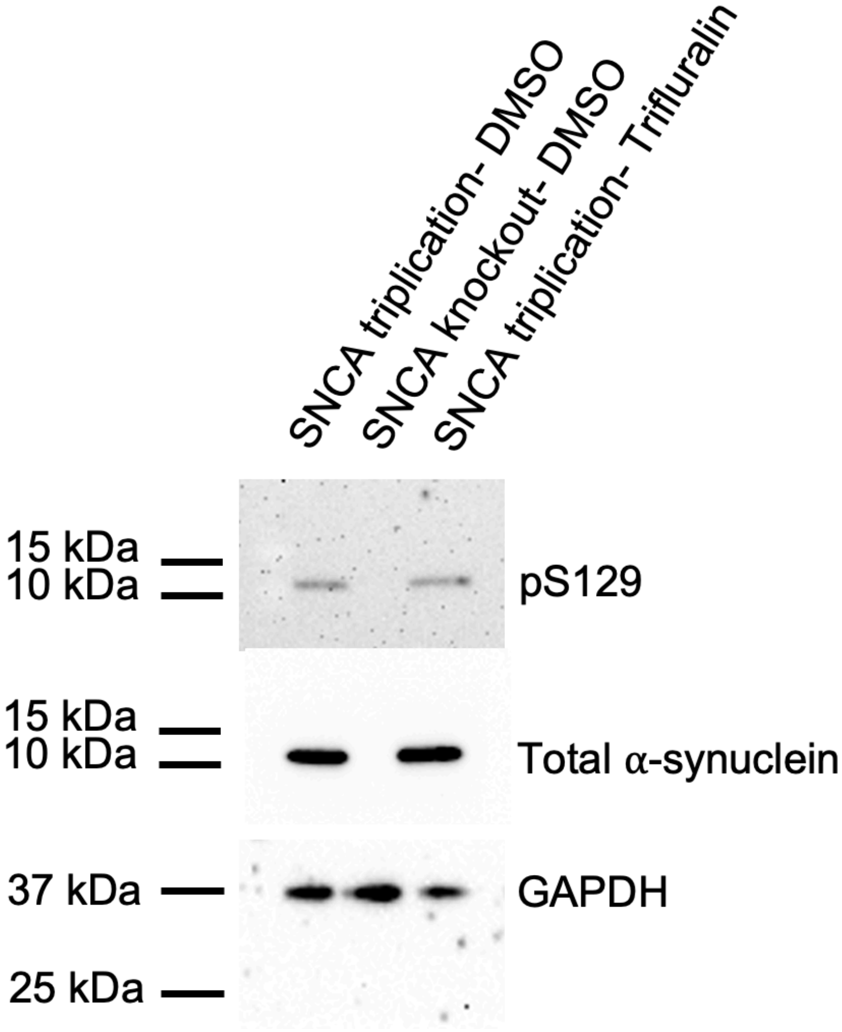

Supplementary Figure 13. Mixed cultures of mDA neurons were treated with sublethal doses of trifluralin or DMSO for 2 weeks and expression of phospho-synuclein (pS129) was measured by Western blot. DMSO treated samples acquired in parallel from an isogenic synuclein knockout line was used to confirm specificity of the antibody for α-synuclein. Representative blots shown. Two additional experiments showed similar results. The upper blot is rabbit anti-pS129, middle blot is total mouse anti- α-synuclein, lower blot is mouse anti-GAPDH after stripping and reprobing of the membrane previously stained for mouse anti α-synuclein. The blots for anti-pS129 and total mouse anti-α-synuclein were derived from a single gel where each sample was loaded into two separate lanes. Following transfer, the blot was cut for parallel processing.

Supplementary References

1. Cockburn M, Mills P, Zhang X, Zadnick J, Goldberg D, Ritz B. Prostate cancer and ambient pesticide exposure in agriculturally intensive areas in California. *Am J Epidemiol* **173**, 1280-1288 (2011).

2. California Department of Water Resources Land Use Surveys. In: *CDWR*) (2013).

3. McElroy JA, Remington PL, Trentham-Dietz A, Robert SA, Newcomb PA. Geocoding addresses from a large population-based study: lessons learned. *Epidemiology* **14**, 399-407 (2003).

4. Figueiredo DM*, et al.* Spatio-temporal variation of outdoor and indoor pesticide air concentrations in homes near agricultural fields. *Atmospheric Environment* **262**, 118612 (2021).

5. Ritz B, Costello S. Geographic model and biomarker-derived measures of pesticide exposure and Parkinson's disease. *Ann N Y Acad Sci* **1076**, 378-387 (2006).

6. Harrer M, Cuijpers P, Furukawa TA, Ebert DD. *Doing meta-analysis with R: a hands-on guide*. Chapman and Hall/CRC (2021).

7. Yu G. clusterProfiler: An universal enrichment tool for functional and comparative study. *BioRxiv*, 256784 (2018).
