# Supplementary material for: A pesticide and iPSC dopaminergic neuron screen identifies and classifies Parkinson-relevant pesticides
Source: Nat Commun. 2023 May 16;14:2803. doi: 10.1038/s41467-023-38215-z (PMC10188516; doi:10.1038/s41467-023-38215-z)
Supplement: Supplementary file 3 — Description of Additional Supplementary Files [file 41467_2023_38215_MOESM3_ESM.pdf]

Description of Additional Supplementary Files

Supplementary Data 1a. Exposure window and pesticide active ingredient application information in epidemiologic PEG study

Supplementary Data 1b. Exposure window and total application information in epidemiologic PEG study, by sex and study wave

Supplementary Data 2. Exposure descriptive, use type, and chemical class information for the pesticides included in the epidemiologic pesticide-wide association study (PWAS).

Supplementary Data 3. PWAS risk estimates, including the overall meta-associations and estimates stratified by study wave and exposure location, for pesticides associated with PD at $p<0.10$.

Supplementary Data 4. Regulatory and toxicity information for the PWAS-implicated pesticides (FDR≤0.10).

Supplementary Data 5. PWAS pesticide class and type overrepresentation analysis (ORA). Comparing overrepresentation of groups in the set of pesticides associated with PD to the expected number based on all analysis pesticides included (N=286). For each pesticide group, odds ratios and 95% CIs were calculated based on frequency information of pesticide association in the different groups, 2-sided p-values were calculated with Fisher's exact test and hypergeometric distribution. We then used an FDR to correct for multiple testing.

Supplementary Data 6. PWAS Sensitivity Analysis 1: Including occupational term (ever worked with pesticides/fertilizers, yes/no) in the model. We conducted univariate, unconditional logistic regression to calculate odds ratios (ORs) and 95% confidence intervals (CIs) for PD with each pesticide (n=288). We combined the OR estimates from each study wave and location in a fixed effects meta-analysis, results shown here. P-values were based on a z-score statistic and two-sided interval. Sensitivity analysis includes additional occupational work history covariate in the model.

Supplementary Data 7. PWAS Sensitivity Analysis 2: Including n=183 study wave 2 controls that completed only abbreviated questionnaire. We conducted univariate, unconditional logistic regression to calculate odds ratios (ORs) and 95% confidence intervals (CIs) for PD with each pesticide (n=288). We combined the OR estimates from each study wave and location in a fixed effects meta-analysis, results shown here. P-values were based on a z-score statistic and two-sided interval. Sensitivity analysis includes additional controls for study wave 2.

Supplementary Data 8. Results from models including a pesticide*sex interaction term. Models with pesticide*sex interaction $p<0.10$ are shown. We conducted univariate, unconditional logistic regression to calculate odds ratios (ORs) and 95% confidence intervals (CIs) for PD for pesticides associated with PD at FDR<0.10 in the primary PWAS analysis and including the interaction term in the model. P-values were based on a z-score statistic and two-sided interval. We then used an FDR to correct for multiple testing.

Supplementary Data 9. Regulatory and toxicity information for the mDA toxic pesticides.

Supplementary Data 10. Pairwise Pearson correlation coefficients for pesticide residential exposure variables for PWAS-implicated pesticides (FDR≤0.10).

Supplementary Data 11. Pairwise Pearson correlation coefficients for pesticide workplace exposure variables for PWAS-implicated pesticides (FDR≤0.10).

Supplementary Data 12. Pesticide clusters determined from hierarchical correlation clustering of residential exposure variables of PWAS-implicated pesticides (FDR≤0.10).

Supplementary Data 13. Pesticide clusters determined from hierarchical correlation clustering of workplace exposure variables of PWAS-implicated pesticides (FDR≤0.10).

Supplementary Data 14. P-values with multiple testing adjustment for individual each of the pairwise comparisons of mDA toxicity for the cotton pesticide combinations. Each combination of two toxicant's THtdTomato count values was compared via Student's t-test, and p-values were adjusted for multiple testing with Benjamini-Hochberg false discovery rate to q-values.

Supplementary Data 15. PEG epidemiologic study population characteristics and exposure window information.
