# Supplementary material for: A pesticide and iPSC dopaminergic neuron screen identifies and classifies Parkinson-relevant pesticides
Source: Nat Commun. 2023 May 16;14:2803. doi: 10.1038/s41467-023-38215-z (PMC10188516; doi:10.1038/s41467-023-38215-z)
Supplement: Supplementary file 5 — Reporting Summary [file 41467_2023_38215_MOESM5_ESM.pdf]

nature portfolio

Corresponding author(s): Kimberly Paul (kimberlp@ucla.edu); Lee Rubin (lee_rubin@harvard.edu); Vikram Khurana (vkhurana@bwh.harvard.edu); Beate Ritz (britz@ucla.edu)

Last updated by author(s): February 10, 2023

Reporting Summary

Nature Portfolio wishes to improve the reproducibility of the work that we publish. This form provides structure for consistency and transparency in reporting. For further information on Nature Portfolio policies, see our Editorial Policies and the Editorial Policy Checklist.

Statistics

For all statistical analyses, confirm that the following items are present in the figure legend, table legend, main text, or Methods section.

n/a	Confirmed	
☐	☒	The exact sample size (n) for each experimental group/condition, given as a discrete number and unit of measurement
☐	☒	A statement on whether measurements were taken from distinct samples or whether the same sample was measured repeatedly
☐	☒	The statistical test(s) used AND whether they are one- or two-sided<br>*Only common tests should be described solely by name; describe more complex techniques in the Methods section.*
☐	☒	A description of all covariates tested
☐	☒	A description of any assumptions or corrections, such as tests of normality and adjustment for multiple comparisons
☐	☒	A full description of the statistical parameters including central tendency (e.g. means) or other basic estimates (e.g. regression coefficient) AND variation (e.g. standard deviation) or associated estimates of uncertainty (e.g. confidence intervals)
☐	☒	For null hypothesis testing, the test statistic (e.g. F, t, r) with confidence intervals, effect sizes, degrees of freedom and P value noted<br>*Give P values as exact values whenever suitable.*
☒	☐	For Bayesian analysis, information on the choice of priors and Markov chain Monte Carlo settings
☐	☒	For hierarchical and complex designs, identification of the appropriate level for tests and full reporting of outcomes
☐	☒	Estimates of effect sizes (e.g. Cohen's d, Pearson's r), indicating how they were calculated

Our web collection on statistics for biologists contains articles on many of the points above.

Software and code

Policy information about availability of computer code

Data collection	Molecular Devices Meta Express Software (version 6.5.3.427) was used for data acquisition in figures 3, 4, 6, Supplementary Figure 5. Nikon Elements (NIS ElementsAR ver. 4.6.0.) and Zeiss Zen software (2012 S4) were also used to acquire images for the cell models.
Data analysis	Statistical analysis of the human population was done using R version 4.1.3 and custom code. The map in Figure 1 was created using ArcGIS; all data graphs in Figure 1, 2, 4, 5, & 6 were created using R and custom code. The network graph in Figure 5B was created using R with custom code and Cytoscape version 3.9.1.<br><br>For the cell models, Columbus (Perkin Elmer, v2.9.1) analysis software was used for image analysis of neurons in Figures 4, 6 and Supplementary Figure 5. Upset plots in Figure 6 were generated in R (Bioconductor) to visualize pesticide combinations on the x-axis (https://jokergoo.github.io/ComplexHeatmap-reference/book/upset-plot.html#upset-making-the-plot). Synergy plots were created from an upset plot of each toxicant combination (ggupset(0.3.0)) and a bar plot of the day 11 neurons' mean THTdTomato brightness (ggplot2 (3.3.5) with a viridis plasma palette (0.5.1).<br><br>For Agilent Seahorse XF Cell Mito stress assay the Wave 2.6.1.53 (2018), Agilent Technologies software was used.<br>To quantify the bands for western blot analysis in the Mitochondrial subunit assays Image Studio Lite Ver 5.2 was used. MoFlo Summit software (version 6.1.16945) and FloJo software (version 10.6.1) were utilized for sorting and representation of cytometry data.<br>For statistical analys of both, western blot and Mito stress assay GraphPad Prism 9 was used.

For manuscripts utilizing custom algorithms or software that are central to the research but not yet described in published literature, software must be made available to editors and reviewers. We strongly encourage code deposition in a community repository (e.g. GitHub). See the Nature Portfolio guidelines for submitting code & software for further information.

Data

Policy information about availability of data

All manuscripts must include a data availability statement. This statement should provide the following information, where applicable:

- Accession codes, unique identifiers, or web links for publicly available datasets
- A description of any restrictions on data availability
- For clinical datasets or third party data, please ensure that the statement adheres to our policy

Source data are provided with this paper. Further data that supports the findings presented in the current study are available from the corresponding authors on reasonable request. .

Human research participants

Policy information about studies involving human research participants and Sex and Gender in Research.

Reporting on sex and gender	We considered gender in the human population-based study of this manuscript. This information was self-reported by participants. In total, our study analyzed data from 907 men and 746 women (shown in Supplemental Table 15). Parkinson's disease is more common among men, therefore our statistical analysis controlled for gender as a covariate. We also provide exposure descriptive information and risk estimates stratified by stratified by gender. Finally, we considered statistical interactions between gender and pesticide exposure which are reported in the manuscript.
Population characteristics	The human epidemiologic study for this manuscript was based on 829 Parkinson's patients and 824 controls. The mean age of the patients was 67.7 (SD=10.6) and 65.9 (SD=11.6) for controls. 76% of the patients and 69% of controls were of European ancestry; 63% of the patients and 47% of the controls were male. This information along with more detailed population characteristics is provided in Supplemental Table 15.
Recruitment	Study enrollment for the epidemiologic study took place over two waves: wave 1 (PEG1): 2000-2007, n=357 PD patients, n=400 population-based controls; wave 2 (PEG2): 2009-2015, n=472 PD patients, n=424 population-based controls. Patients were first (PEG1) identified through large medical groups, neurologists, and public service announcements and second (PEG2) from the pilot PD registry program in California. Population-based controls for both study waves were required to be > 35 years of age, have lived within one of the three counties the patients resided in (Kern, Fresno, Tulare) for at least 5 years before enrollment, and not have a diagnosis of PD. We identified potentially eligible controls initially through Medicare enrollee lists (2001) but mainly from publicly available residential tax-collector records (after 2001 due to HIPAA restrictions). We used two sampling strategies for controls to increase enrollment success and representativeness of the source population: a) for PEG1, random selection from the Medicare enrollee lists and of residential parcels (identified from the tax-collector records) followed by mail or phone enrollment, and b) for PEG2, random selection of clustered households (five per cluster, identified through the tax-collector records) visited in person to enroll at least one eligible control from each cluster (only one per household allowed). A detailed description of all enrollment numbers is provided in the supplemental methods.
Ethics oversight	The PEG study was approved by the UCLA Institutional Review Board (IRB#21-000256 and IRB#11-001530) and informed consent was obtained from all individuals. Our research conformed to the Declaration of Helsinki.

Note that full information on the approval of the study protocol must also be provided in the manuscript.

Field-specific reporting

Please select the one below that is the best fit for your research. If you are not sure, read the appropriate sections before making your selection.

☒ Life sciences ☐ Behavioural & social sciences ☐ Ecological, evolutionary & environmental sciences

For a reference copy of the document with all sections, see nature.com/documents/nr-reporting-summary-flat.pdf

Life sciences study design

All studies must disclose on these points even when the disclosure is negative.

Sample size	For the human population-based analysis, the sample size was based on all available data from the PEG studies (n=1653).<br><br>For the cell models, sample size was based on plating density of neurons for the experiments. Four fields of live images were acquired with a 10x objective typically resulted in sampling 60-120 neurons per well depending on the assay. Sample size estimates were derived from preliminary experiments using a positive control (rotenone) to determine how many biological replicates and how many cells needed to be measured to detect an observed difference in survival.
Data exclusions	Data were not excluded from analysis.
Replication	For the human population-based analysis, we used two study waves of the PEG population. These study waves are independent populations,

	recruited from the same geographic area, with the same study protocol (neurologic exam, patient questionnaire, exposure assessment, etc). This allowed us to assess replication from one study wave to the other.<br><br>For the cell models, 2-3 technical replicates were used for each pesticide for the screen described in Figure 4. The screen in Figure 4 was performed once. The combinatorial treatments described in Figure 6 represent n=4. Biological replicates are specified in the text of figure legends or results as appropriate.
Randomization	For the human population-based analysis, we conducted analysis of existing data from an observational study. The study subjects were not randomly assigned to exposure groups, but compared based on pesticide use reports detailing how much pesticide was applied near the homes and workplaces of participants since 1974.<br><br>For the cell models, the same iPSC line was utilized for all described experiments. Constraints of screening library construction did not permit well to well randomization in data for Figure 4. Well treatments were randomized for Figure 6 using HP Digital Dispenser software.
Blinding	For the human population-based analysis, we conducted analysis of existing data from an observational study. Thus blinding was not relevant. For the cell models, blinding was not performed in cell quantitation experiments due to the use of image analysis algorithms (Columbus software) applied equally across all wells of an experiment.

Reporting for specific materials, systems and methods

We require information from authors about some types of materials, experimental systems and methods used in many studies. Here, indicate whether each material, system or method listed is relevant to your study. If you are not sure if a list item applies to your research, read the appropriate section before selecting a response.

Materials & experimental systems

n/a	Involved in the study
☐	☒ Antibodies
☐	☒ Eukaryotic cell lines
☒	☐ Palaeontology and archaeology
☒	☐ Animals and other organisms
☒	☐ Clinical data
☒	☐ Dual use research of concern

Methods

n/a	Involved in the study
☒	☐ ChIP-seq
☐	☒ Flow cytometry
☒	☐ MRI-based neuroimaging

Antibodies

Antibodies used	Primary ABs<br>Total OXPHOS Rodent WB Antibody Cocktail [supplier: abcam inc., cat#: ab110413, Lot#: 2101002618]<br>SDHA Monoclonal Antibody, dilution 1:10,000 (Clone: 2E3GC12FB2AE2) [supplier: Fisher Scientific, cat#: 459200 Lot#: WH3388949]<br>Anti-TOMM20 antibody produced in rabbit, dilution 1:1,000 [supplier: Sigma Aldrich, cat#: HPA011362, Lot#: 317737]<br>Anti-Actin antibody produced in rabbit, dilution 1:1,200 [supplier: Sigma Aldrich, cat#: A2066, Lot#: 099M-4864V]<br>Sheep anti-TH, dilution 1:500 [supplier: Pel-Freeze #960101]<br>Rabbit anti-RFP, dilution 1:500 [supplier: Rockland 600-401-379]<br>Rabbit anti-phospho-Ser-129, dilution 1:1,000 [supplier: Abcam Cat. # ab51253]<br>Mouse anti-Alpha-Synuclein (Syn1), dilution 1:1,000 [Clone: 42; supplier: BD Transduction Laboratories Cat. # 610787]<br>Mouse anti-GAPDH, dilution 1:15,000 [Clone 6C5; supplier: EMD Millipore Cat. # MAB374]<br><br>Secondary AB<br>Donkey anti-Mouse IgG H&L, dilution 1:10,000 (IRDye® 680RD) preadsorbed [supplier: LI-COR inc., cat#: ab216778, Lot#: GR3408325-1]<br>Goat anti-Rabbit IgG H&L, dilution 1:10,000 (IRDye® 800CW) preadsorbed [supplier: abcam inc., cat#: ab216773, Lot#: GR3408322-1]<br>Donkey anti-Sheep IgG (H+L) Highly Cross-Adsorbed Secondary Antibody, Alexa Fluor 488, dilution 1:500 [Life Technologies # A11015]<br>Donkey anti-Rabbit IgG (H+L) Highly Cross-Adsorbed Secondary Antibody, Alexa Fluor 594, dilution 1:500 [Life Technologies # A21207]<br>Goat anti-Mouse IgG Cross-Adsorbed Secondary Antibody, HRP, dilution 1:10,000 [Bio-Rad 170-6516]<br>Goat anti-Rabbit IgG Secondary Antibody, HRP, dilution 1:10,000 [Bio-Rad 170-6515]
Validation	Sheep anti-TH: From manufacturer website/datasheet: "The antibody has been tested in Western blots of SDS-solubilized human and mouse striatal samples and in IHC applications with monkey (Macaque) brain sections. References:<br>1) Kish SJ et al. (2001) Neuropsychopharmacology 24:561-567.<br>2) Salvatore MF et al. (2001) J Neurochem 79:349- 360.<br>3) Witkovsky P et al. (2000) J Chem Neuroanat 19:105-116.<br>4) Zhu MY et al. (2000) J Neurosci Meth 99:37-44.<br>5) Zhu MY et al. (1999) Biol Psychiatry 46:1275-1286."<br><br>Rabbit anti-RFP: From manufacturer's website: "This product was prepared from monospecific antiserum by immunoaffinity

chromatography using Red Fluorescent Protein (Discosoma) coupled to agarose beads followed by solid phase adsorption(s) to remove any unwanted reactivities. Expect reactivity against RFP and its variants: mCherry, tdTomato, mBanana, mOrange, mPlum, mOrange and mStrawberry. Assay by immunoelectrophoresis resulted in a single precipitin arc against anti-Rabbit Serum and purified and partially purified Red Fluorescent Protein (Discosoma). No reaction was observed against Human, Mouse or Rat serum proteins."

Anti-phospho-Ser-129: From manufacturer's website- "This antibody only detects alpha synuclein phosphorylated on Ser129. IHC-P: This antibody showed no staining in human hippocampus normal brain and showed staining in Parkinson's brain as expected." Manufacturer's website also shows Western Blot of in vitro kinase activity detecting phopsho S129 only in the presence of PLK2 and ATP.

Anti-Alpha-Synuclein (Syn1): Published work shows immunohistochemical staining seen in transgenic mice expressing human synuclein that is absent in wild type mice (PMID: 10934251). Supplemental figure 11C of this manuscript shows absence of staining in neurons derived from an alpha synuclein knockout iPSC line.

Anti-GAPDH: From manufacturer's website. Recognizes "Glyceraldehyde-3-phosphate dehydrogenase (GAPDH) from skeletal muscle. Antibody also recognizes cardiac GAPDH. GAPDH enzyme is detected in many non-muscle cells lines including HeLa, HCT-116 cells, U937 and THP-1 cells among others… [species reactivity includes] Human, porcine, canine, rat, mouse, rabbit, cat, and fish skeletal muscle. It has been reported that this antibody does not react with GAPDH from E. coli." Manufacturer's website provides technical data sheet which depicts Western Blot validation results from eight distinct cell lines showing the same band a the expected molecular weight.

Total OXPHOS Rodent WB Antibody Cocktail: This is a cocktail of 5 mouse mAbs. "One each against CI subunit NDUFB8 (ab110242), CII-30kDa (ab14714), CIII-Core protein 2 (ab14745), CIV subunit I(ab14705) and CV alpha subunit (ab14748) as an optimized premixed cocktail." Specificity data for these antibodies are as follows:
CI subunit NDUFB8: From manufacturer online documentation, "Skeletal muscle immunohistochemistry using ab110242 on frozen tissue sections from a patient with a single large deletion of the mtDNA show a mosaic of complex I positive and complex I negative fibers."
CII-30kDa: Manufacturer website demonstrates "ab14714 staining of SDHB in wild-type HEK293 cells and SDHB knockout HEK293 cells". Images demonstrate absence of staining in the knockout HEK293 cells.
CIII-Core protein 2: Manufacturer's website demonstrates "Mitochondrial localization of complex III visualized by immunofluorescence using anti-complex III subunit Core 2 mAb 2E3GC12FB2AE2 (ab14745)....[in] Cultured human embryonic lung-derived fibroblasts (strain MRC5)."
CIV subunit I: Figure 3E of Zierz et al (PMID: 31167410) demonstrates destabilization and decreased protein abundance of MT-CO1 (as a part of complex 4) in the setting of MT-CO2 deficiency. Manufacturer's website also demonstrates mitochondrial localization by immunocytochemistry.
CV alpha subunit: Manufacturer's website identifies "isolated mitochondria from human, cow, rat and mouse heart. Human liver tissue lysate. HepG2 whole cell lysate" as positive controls. All samples produce a prominent band at the same size for each source of isolated mitochondria. Immunocytochemistry of ab14748 stained MCF7 (Human breast adenocarcinoma cell line) cells shows mitochondrial staining pattern.

SDHA Monoclonal Antibody (Clone: 2E3GC12FB2AE2): SDHA Antibody (459200). From manufacturer's website: Antibody specificity was demonstrated by siRNA mediated knockdown of target protein. SH-SY5Y cells were transfected with SDHA siRNA and decrease in signal intensity was observed in Western Blot application using Anti-SDHA Monoclonal Antibody.

Anti-TOMM20 antibody: Per the manufacturer's website: "Anti-TOMM20 antibody produced in rabbit, a Prestige Antibody, is developed and validated by the Human Protein Atlas (HPA) project . Each antibody is tested by immunohistochemistry against hundreds of normal and disease tissues. These images can be viewed on the Human Protein Atlas (HPA) site"

Anti-Actin antibody: From the manufacturer's website, "In immunoblotting, the product localizes actin in many species ranging from human skeletal muscle to amoeba. The product recognizes the 42 kDa actin band using immunoblotting techniques in human or animal tissue extracts." The antibody has also been subject to Sigma's "enhanced validation" criteria with independent confirmation of staining patterns in multiple tissues when compared to multiple other anti-actin antibodies.

Eukaryotic cell lines

Policy information about cell lines and Sex and Gender in Research

Cell line source(s)

The synuclein triplication iPSC line was generated from the fibroblasts of a male subject from the "Iowa Kindred" with rapidly progressive parkinsonism presenting at age 41. Additional description in Chung, Khurana 2013 (DOI: 10.1126/science.1245296). The wild-type (2-copy) synuclein iPSC line is the mutation corrected control of another iPSC line. The original iPSC line was generated from fibroblasts of a symptomatic male subject of previously described kindred (Zarranz et al. Ann Neurol 2004;55:164–173). The subjects shows indicators of Parkinson's Disease in both central nervous system (illustrated by DAT-scan showing degeneration of nigra-striatal pathway) and peripheral nervous system (accumulation of phosphorylated alpha synuclein can be found in sympathetic neurons by skin biopsy). Mutation correction was performed using a CRISPR/Cas9 and piggybac system (Woodard, L.E., and Wilson, M.H. 2015. Doi: 10.1016/j.tibtech.2015.06.009).

Authentication

G-banded karyotyping confirmed a normal male karyotype before and after genetic manipulations.

Mycoplasma contamination

These cell lines (iPSCs and resultant neurons) were tested at least monthly for the presence of mycoplasma and were negative.

Commonly misidentified lines
(See ICLAC register)

No commonly misidentified lines were used in this work.

Flow Cytometry

Plots

Confirm that:

- [x] The axis labels state the marker and fluorochrome used (e.g. CD4-FITC).
- [x] The axis scales are clearly visible. Include numbers along axes only for bottom left plot of group (a 'group' is an analysis of identical markers).
- [x] All plots are contour plots with outliers or pseudocolor plots.
- [x] A numerical value for number of cells or percentage (with statistics) is provided.

Methodology

Sample preparation	Spheres are collected in a 15mL conical tube from suspension culture plate, washed with PBS and resuspended in 2mL of 0.25% Trypsin EDTA with 25ng/mL of DNASe added prior to incubation at 37 degrees in water bath or rotating shaker for 5-7 minutes. 500uL of FBS is then added to stop the reaction. Following a PBS wash, the EBs are triturated 5-7 times with a P1000 in a trituration solution (PBS with 5% FBS, 25mm Glucose, 1x glutamax). Cells are then washed with PBS and pelleted at 300 x g for 5 minutes. Washes are repeated 3-5 times prior to plating or FACS sorting. Large clumps and aggregates are filtered using a 35µM CellTrics filter. Y-27632 at 10µM is present for sorting and collection in differentiation media.
Instrument	A MoFlo Astrios and MoFlo XDP (Beckman-Coulter, both equipped with 100um nozzle at 30psi) were used to sort.
Software	Summit Software version 6.1.16945; FloJo software (version 10.6.1)
Cell population abundance	The brightest 30-40% of cells are included in order to minimize non-neuronal cell types or immature/neuronal progenitors expressing a low level of the THtdTomato reporter. Initial, extensive characterization of the THTdtomato reporter is described in Ahfeldt et al 2020 (DOI: 10.1016/j.stemcr.2019.12.005).
Gating strategy	Forward scatter (FSC) and side scatter (SSC) were used to eliminate debris in conjunction with FSC-width (FSC-W) and SSC-area (SSCA) to focus on single cells. Cells were then gated on exclusion of Sytox Red dye exclude dead cells. TdTomato fluorescence vs. GFP fluorescence was used to select THtdT+ cells. The green channel was used for auto-fluorescence.

- [x] Tick this box to confirm that a figure exemplifying the gating strategy is provided in the Supplementary Information.
